# Tumor Secretome to Adoptive Cellular Immunotherapy: Reduce Me Before I Make You My Partner

**DOI:** 10.3389/fimmu.2021.717850

**Published:** 2021-08-10

**Authors:** Mikel Etxebeste-Mitxeltorena, Inés del Rincón-Loza, Beatriz Martín-Antonio

**Affiliations:** Department of Experimental Hematology, Instituto de Investigación Sanitaria-Fundación Jiménez Diaz, UAM, Madrid, Spain

**Keywords:** tumor secretome, SASP, senescence, immunotherapy, macrophages, CAR-T cells, NK cells, T cells

## Abstract

Adoptive cellular immunotherapy using chimeric antigen receptor (CAR)-modified T cells and Natural Killer (NK) cells are common immune cell sources administered to treat cancer patients. In detail, whereas CAR-T cells induce outstanding responses in a subset of hematological malignancies, responses are much more deficient in solid tumors. Moreover, NK cells have not shown remarkable results up to date. In general, immune cells present high plasticity to change their activity and phenotype depending on the stimuli they receive from molecules secreted in the tumor microenvironment (TME). Consequently, immune cells will also secrete molecules that will shape the activities of other neighboring immune and tumor cells. Specifically, NK cells can polarize to activities as diverse as angiogenic ones instead of their killer activity. In addition, tumor cell phagocytosis by macrophages, which is required to remove dying tumor cells after the attack of NK cells or CAR-T cells, can be avoided in the TME. In addition, chemotherapy or radiotherapy treatments can induce senescence in tumor cells modifying their secretome to a known as “senescence-associated secretory phenotype” (SASP) that will also impact the immune response. Whereas the SASP initially attracts immune cells to eliminate senescent tumor cells, at high numbers of senescent cells, the SASP becomes detrimental, impacting negatively in the immune response. Last, CAR-T cells are an attractive option to overcome these events. Here, we review how molecules secreted in the TME by either tumor cells or even by immune cells impact the anti-tumor activity of surrounding immune cells.

## Introduction

Today, it is widely recognized that chronic inflammation is a driver of cancer (1), being estimated that 15-20% of cancers are inflammation-related (2). This association has been observed in different contexts, such as persistent *Helicobacter pylori* infection or autoimmune diseases like inflammatory bowel disease that increase the risk of developing gastric cancer (3) or colorectal cancer (4), respectively. Numerous studies have found associations of inflammatory markers with a higher risk of developing cancer. For instance, 15% of patients with cardiovascular disease, after a median follow-up of 8.3 years, developed different types of cancer whose incidence was associated with high C-reactive protein (CRP) levels (5). In addition, IL6 levels are also associated with an increased risk of developing different types of cancer (6). Moreover, IL1β inhibition reduced CRP and IL6 levels and the incidence of developing lung cancer in patients with atherosclerosis who had a myocardial infarction (7).

Both immune and tumor cells promote this pro-inflammatory microenvironment. Expressly, tumor cells release a secretome that displays an altered composition compared to the normal tissue from which they are derived (8). This secretome contains cytokines, chemokines, hormones, metabolites, and growth factors involved in cell-cell communication, angiogenesis, hypoxia, metastasis, extracellular matrix remodeling, and drug resistance (8, 9), where tumor cells employ it as a mechanism of immune evasion (10–12). On the other side, the different subsets of immune cells will also release immunosuppressive and inflammatory factors that will shape the tumor microenvironment (TME), promoting or inhibiting cancer progression (13).

The anti-tumor activity of immune cells infiltrating tumors led to the development of adoptive cellular immunotherapy administering natural killer (NK) cells, T cells, or genetically modified chimeric antigen receptor (CAR)-T cells in cancer patients (14–17). Clinical results administering different immune cells have been reviewed by others (**Table 1**). However, despite promising results in these studies for some malignancies (26), immune cells do not persist long for other malignancies, and patients end up relapsing (27). Once immune cells achieve the tumor, they will have to face tumor cells and their secretome that may polarize their anti-tumor activity to a pro-tumoral one, increasing angiogenesis and enhancing tumor growth (28). Moreover, after chemotherapy treatment, tumor cells can reach a senescent state, known as therapy-induced senescence (TIS), that shapes the tumor secretome to a variety of pro-inflammatory and angiogenic proteins known as “senescence-associated secretory phenotype” (SASP). The SASP may enhance the immune response at initial stages and contribute to a favorable environment for tumor growth at late stages (29). For example, senescent fibroblasts, much more than pre-senescent fibroblasts, secrete VEGF that causes premalignant and malignant epithelial cells to form tumors, suggesting that although cellular senescence suppresses tumorigenesis early in life, it may also promote cancer (30).

**Table 1:** Reviews indicating clinical results with different types of immune cells administered in immunotherapy studies in cancer patients.

Types of immune cell administered	Reference
NK cells	(18, 19)
CAR-T cells	(20–22)
TILs:	(23–25)

NK, natural killer; CAR, chimeric antigen receptor; TILs, tumor infiltrating lymphocytes.

Here, we review how the tumor secretome can shape the immune response achieving a state when immune cells no longer recognize tumor cells and instead, they secrete proteins that breed the TME. We will specifically focus on the impact on T cells, CAR-T cells, and NK cells, which are currently used in adoptive cellular immunotherapy (14–17, 31), and macrophages due to their relevant role in removing dying/senescent tumor cells after cancer treatment (32). The impact of these molecules is summarized in **Table 2**. Moreover, we will review the effect of the tumor secretome in the immune response when tumor cells become senescent due to chemotherapy treatments.

**Table 2:** Impact of secreted factors in the tumor microenvironment (TME) over the different immune cell populations and description of receptors acting as eat me or don’t eat me signals for phagocytic activity of macrophages.

Factor	Type of cell	Effect	Reference
TGFβ	CD8	Suppresses IFN-γ production	(33)
	Th1	Suppresses IFN-γ production and induces differentiation to T-reg and Th17 cells.	(34, 35)
	PB-NK	Converts cytotoxic CD56^dim^ and CD56^bright^ PB-NK cells into dNK-like cells.	(36, 37)
		Added to IL15 and IL18 the effects are enhanced.	(38)
	PB-NK	Down-regulates NKP30, NKG2D and DAP10 and, consequently, NKG2D.	(39, 40)
	PB-NK	At low doses up-regulates CXCR4 and CXCR3. At high doses, down-regulates NKp30, limiting NK killer activity.	(41)
	PB-NK	In combination with hypoxia and 5-aza-2′-deoxycytidine polarizes PB-NK cells to dNK-like cells.	(37)
IL10	APCs	Down-regulates HLA-II on APCs inhibiting antigen presentation.	(42)
	CD8	Induces intratumoral antigen presentation with infiltration and activation of CD8 T cells expressing IFNγ and granzymes.	(43)
HLA-G	CD8	Up-regulates CTLA-4, PD-1, TIM-3, and CD95.	(44)
IFNγ	Tumor cells	PD-L1 up-regulation.	(45)
FGL1	CD8	LAG-3 up-regulation with T cell inhibition.	(46)
Gal-9	Th1	Loss of IFNg producing cells and suppression of Th1 autoimmunity.	(47)
Nectin-3	T cells and monocytes	Promote lymphocyte transmigration through interaction with Nectin-2 on endothelial cells.	(48)
Nectin-2	T cell	T cell homing migration to the spleen through TIGIT interaction.	(49)
	PB-NK	Binds to TIGIT inhibiting NK cell cytotoxicity.	(50)
PVR	PB-NK	Binds to TIGIT inhibiting NK cell cytotoxicity.	(50)
PGE2	CD8	Suppression of activity.	(51)
	CD4	Suppression of Th1 activity and promotion of Th2, Th17 and T-reg.	(51)
	PB-NK	In thyroid cancer and melanoma inhibits NKG2D, NKp44, NKp30, and TRAIL suppressing NK cell cytotoxicity.	(10, 52)
	PB-NK	In melanoma down-regulates NKp44 and NKp30 leading to NK cell inhibition.	(53)
	Macrophages	Reduction of CCL5 production.	(54)
IDO	CART-19	Inhibition of CART cell activity.	(55)
Lactic acid	CD8	Suppresses nutrient uptake leading to impaired activation.	(56)
	NK	Suppresses nutrient uptake leading to impaired activation.	(56)
Glycodelin-A	CD56 ^bright^ PB-NK	Polarizes CD56^bright^ into dNK-like cells.	(57)
HLA-G	PB-NK	Induction of senescence with SASP secretion promoting vascular remodeling and angiogenesis.	(58)
Hypoxia	T cells	Favors a glycolytic metabolism and increased lactate production, dampening T effector functions.	(59)
	PB-NK	Avoids the ability to upregulate NKp46, NKp30, NKp44, and NKG2D in response to activating cytokines.	(60)
	PB-NK	Degrades NK cell granzyme B by autophagy.	(61)
	PB-NK	Reduced ability to release IFNγ, TNFα, GM-CSF, CCL3, and CCL5, and preservation of immature CD56^bright^ NK cells expressing CCR7 and CXCR4, resembling dNK-like cells.	(62)
	Macrophages	Activates granulin expression in macrophages through VEGF, conferring increased angiogenic potential.	(63)
	Macrophages	In pancreatic cancer promotes release of exosomes containing miR-301a-3p that induce M2 polarization.	(64)
	Macrophages	Induces CXCL12 and CXCR4 expression, which modulate the migration of monocyte-derived macrophages, and TAMs.	(65)
IL6	Macrophage	Induces M2 polarization in colorectal cancer models.	(66)
OSM	Macrophage	M2 polarization *via* mTOR signaling complex 2-Akt1.	(67)
CCL2	Macrophage	Recruitment of M1 to polarize them to metastasis-associated macrophages.	(68)
IL34	Macrophage	Increase recruitment of M2 macrophages in osteosarcoma.	(69)
VEGF-A	Macrophage	With IL10 and IL4 secreted by tumor cells and macrophages, respectively, induced M2 polarization.	(70)
Versican	Macrophage	Activates macrophages to release TNFα enhancing growth of tumor cells.	(71)
MIF	Macrophage	Recruitment of macrophages through TGFβ secretion by Kupffer cells that creates a fibrotic microenvironment.	(72)
ST2	Macrophage	M1 macrophage polarization in models of lung cancer.	(73)
miR-21	Macrophage	Polarization of monocytes to M2 macrophages, secretion of IL6, IL8, CCL2, and CCL5.	(74)
CD47	Macrophage	In tumor cells is a don’t eat me signal for macrophages.	(75)
PD-1	Macrophage	Don’t eat signal in macrophages.	(76)
β2M subunit (HLA-I)	Macrophage	In tumor cells is a don’t eat me signal for macrophages through interaction with LILRB1.	(77)
CD24	Macrophage	In tumor cells is a don’t eat me signal for macrophages.	(78)

PB-NK, peripheral blood NK cells; dNK, decidual NK cells; T-reg, regulatory T cell; APCs, antigen presenting cells; IFN-γ, interferon-γ; TGFβ, transforming growth factorβ; FGL1, fibrinogen-like 1; GAL-9, galectin-9; IL, interleukin; HLA, human leukocyte antigen; miR, microRNA; OSM, oncostatin-M; VEGF, vascular endothelial growth factor; MIF, macrophages migration inhibitory factor; ST2, suppression of tumorigenicity 2.

## Impact of Tumor Secretome in the Anti-Tumor Activity of Immune Cells

### T Cells

Tumor cells with stromal cells, endothelial cells, fibroblasts, and immune cells create a suitable TME that favors tumor progression (79–81). The ability of T cells to infiltrate this TME has led to the development of adoptive cellular immunotherapy to treat cancer patients with tumor-infiltrating lymphocytes (TILs) or CAR-T cells (14, 15, 31). Interestingly, the TME can shape the anti-tumor activity of T cells depending on a variety of secreted molecules. We detail here the impact of some of these released factors.

**Figure 1:**
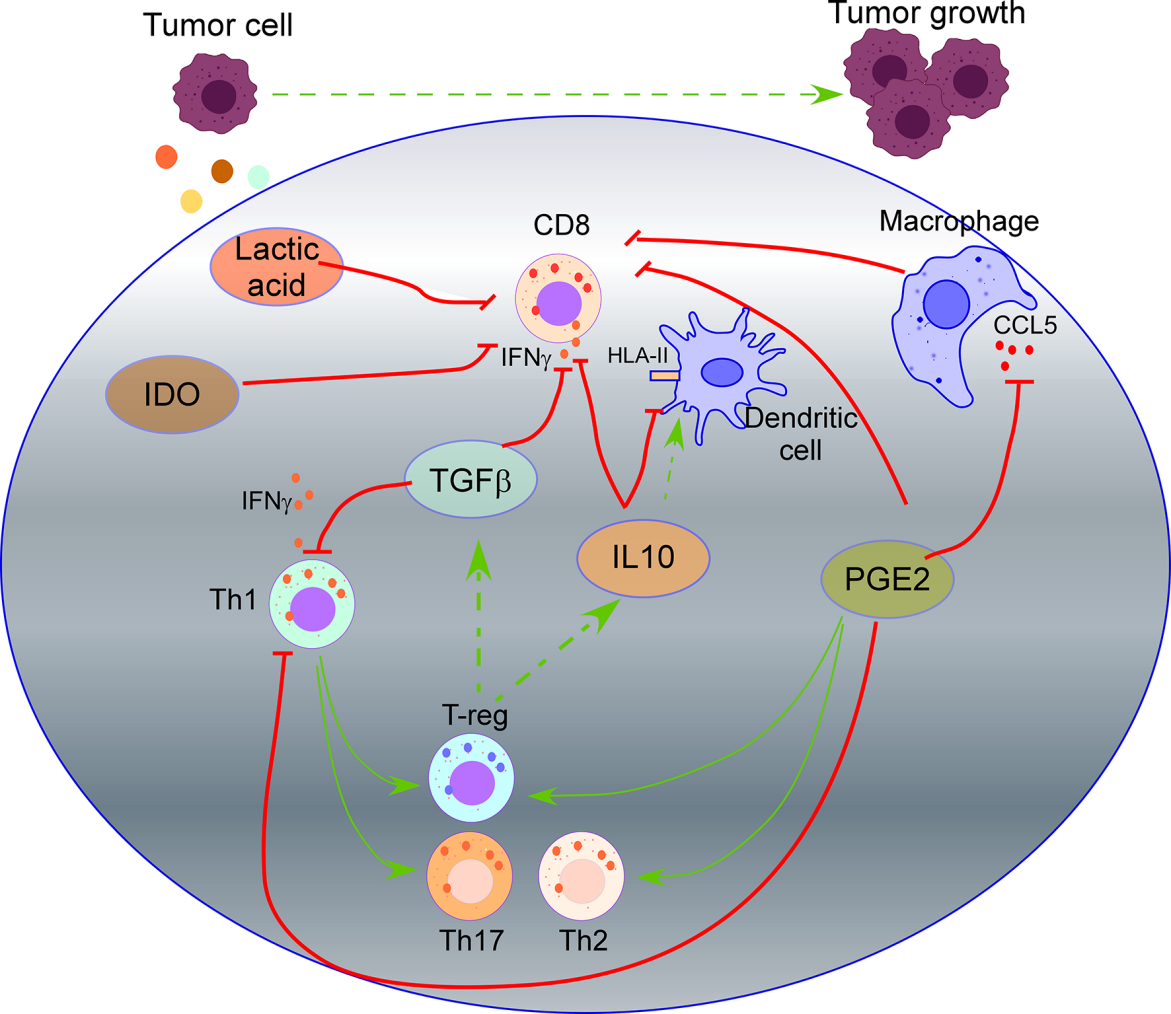
Impact of tumor secretome in T cell activity. TGF-β secreted by tumor cells suppresses IFN-γ production by Th1 and effector CD8 T cells, inducing the differentiation of CD4 T cells to regulatory (T-reg) cells and Th17 cells. T-reg cells also release TFG-β and IL10 that will suppress the activation of CD8 T cells. IL10 secreted by tumor cells down-regulates HLA-II on dendritic cells, inhibiting antigen presentation. Prostaglandin 2 (PGE2) secreted by tumor cells suppresses the functions of CD8 T cells and Th1 cells, and promotes Th2, Th17, and T-reg cell response. PGE2 reduces CCL5 production by macrophages, which is required for T cell proliferation. Secretion of Indoleamine 2,3 dioxygenase (IDO) by tumor cells produces metabolites that inhibit T cell activity. Lactic acid produced by tumor cells suppresses nutrient uptake by CD8 T cells.

TGF-β, a highly recognized immunosuppressive cytokine secreted by tumor cells (33), suppresses IFN-γ production by Th1 and effector CD8 T cells, inducing the differentiation of CD4 T cells to both regulatory (T-reg) cells and Th17 cells. T-reg cells that also release TFG-β and IL10 will further suppress the activation of CD8 T cells, promoting tumor cell growth (34, 35). IL10 production by tumor cells down-regulates HLA-I and HLA-II on tumor cells and HLA-II on antigen-presenting cells (APCs), inhibiting antigen presentation becoming an escape mechanism from immune surveillance (42, 82–84). On the other side, cancer models have shown that IL10 also induces intratumoral antigen presentation with infiltration and activation of tumor-specific cytotoxic CD8 T cells expressing IFNγ and granzymes (43) (**Figure 1**).

**Figure 2:**
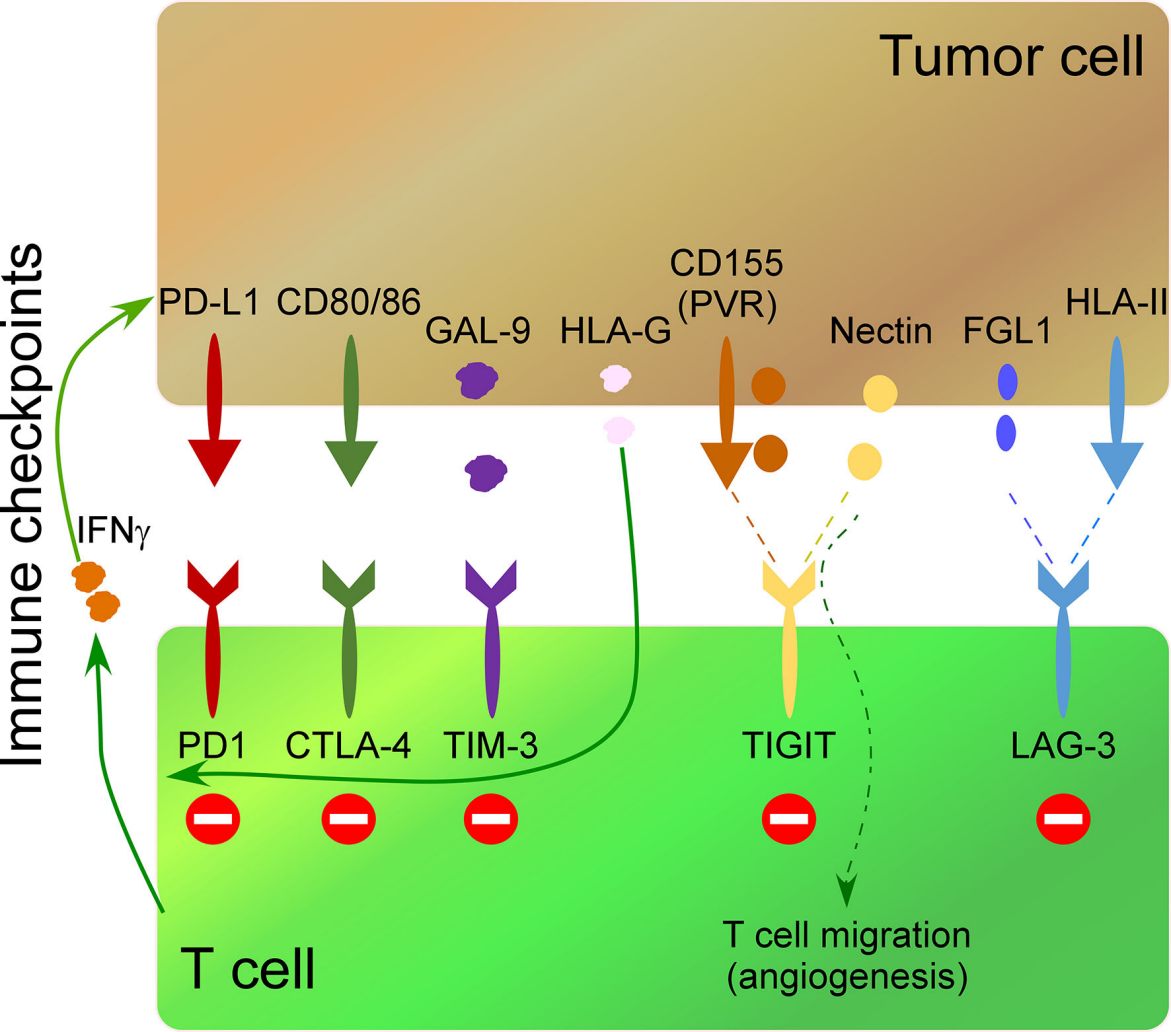
Impact of some secreted molecules in the TME on the expression of immunocheckpoints in T cells. The most common immune checkpoints on T cells include programmed death 1 (PD-1), cytotoxic T lymphocyte antigen 4 (CTLA-4), T cell immunoglobulin and mucin-3 (TIM-3), T cell immunoglobulin and ITIM domain (TIGIT) and lymphocyte activation gene 3 (LAG3), which interact with their ligands on tumor cells. IFNγ release by activated T cells induces PD-L1 up-regulation in tumor cells. TIM-3 interaction on Th1 cells with Galectin-9 (Gal-9) on tumor cells inhibits Th1 cell responses. Soluble HLA-G released by tumor cells up-regulates PD-1, CTLA-4, and TIM-3, on T cells. CD155 (PVR), and the Nectin family are ligands of TIGIT. Soluble PVR is released by tumor cells. Soluble Nectins released by cancer cells mediate transendothelial migration of immune cells promoting angiogenesis. HLA-II over-expression by tumor cells and fibrinogen-like 1 (FGL1) secreted by tumor cells impact the expression of LAG-3 in T cells.

A wide field of research in cancer immunotherapy consists of inhibiting immune-checkpoint receptors on immune cells and their ligands in tumor cells. The interaction of these receptors/ligands modulates the activity of immune cells to limit the development of auto-immunity and create immunotolerant T cells. Therefore, the inhibition of these interactions with monoclonal antibodies increases their anti-tumor activity. The most common immune checkpoints include cytotoxic T lymphocyte antigen 4 (CTLA-4), programmed death 1 (PD-1), T cell immunoglobulin and mucin-3 (TIM-3), B and T lymphocyte attenuator (BTLA), lymphocyte activation gene 3 (LAG3), adenosine 2A receptor (A2AR) and T cell immunoglobulin and ITIM domain (TIGIT) (85–91). Secreted molecules by tumor cells impact the expression of immune-checkpoint receptors on immune cells. For instance, release of soluble HLA-G by tumor cells up-regulates CTLA-4, PD-1, TIM-3, and CD95 on CD8 T cells impacting their anti-tumor activity (44). On the other hand, cytokines released by activated immune cells can up-regulate ligands of immune-checkpoints in tumor cells. Thus, IFNγ release by activated T cells induces PD-L1 up-regulation in tumor cells (45) (**Figure 2**).

HLA-II over-expression by tumor cells (92) and fibrinogen-like 1 (FGL1), a protein secreted by liver cells and tumor cells (46), are ligands of LAG-3, and their secretion impact the expression of LAG-3 in T cells, promoting an immunosuppressive function. TIM-3 is expressed on Th1 cells, and its interaction with its ligand Galectin-9 (Gal-9) on tumor cells inhibits Th1 cell responses (47) (**Figure 2**). Both overexpression of Gal-9 on gastric cancer cells and expression of TIM-3 on immune cells correlates negatively with poor outcomes in cancer patients (93) and lead to an increase in granulocytic myeloid-derived suppressor cells that inhibit immune responses impacting tumor growth (94).

TIGIT ligands include CD155 (PVR), and the Nectin family (95, 96) (**Figure 2**), which are over-expressed in many human malignancies (97). Specifically, soluble PVR is a valuable biomarker for cancer development, where higher soluble PVR levels are detected in lung, gastrointestinal, breast, and gynecologic cancers compared to healthy donors, being even higher at advanced stages of the disease (98). Of interest, Nectins promote the transendothelial migration of cells and associate with poor prognosis and advanced disease stages in different types of cancer (99). Soluble Nectin-4 released by cancer cells interacts with integrin-β4 on endothelial cells, promoting angiogenesis (100). Of interest, Nectins also mediate transendothelial migration of immune cells (48). For instance, Nectin-2 promotes endothelial cell migration, endothelial tube formation, and T cell homing migration to the spleen, promoting an angiogenic function (49); Nectin-3 expressed by T cells and monocytes binds to endothelial cells through Nectin-2 promoting the transmigration of immune cells (48). This angiogenic function of soluble Nectins released by tumor cells suggests an essential role of the tumor secretome polarizing the cytotoxic activity of T cells to an angiogenic one.

Prostaglandin 2 (PGE2) is a crucial mediator of immunopathology in chronic infections and cancer. PGE2 secreted by tumor cells suppresses the effector functions of CD8 T cells and Th1 cells, promotes Th2, Th17, and T-reg cell response, and inhibits the attraction of immune cells (51). Moreover, PGE2 reduces CCL5 production by macrophages (54), which is required for IL2, IFN-γ production, and T cell proliferation (101). Recent studies revealed that COX2/mPGES1/PGE2 pathway in tumor cells up-regulates PD-L1 in tumor-associated macrophages (TAMs) and myeloid-derived suppressor cells (MDSCs), which is followed by T cell elimination (102).

In addition, the tumor secretome impacts the metabolic activity of T cells through the competitive removal of essential nutrients for T lymphocytes. In this sense, secretion of “Indoleamine 2,3 dioxygenase” (IDO), which catalyzes tryptophan degradation, produces metabolites that inhibit T cell activity. In a murine lymphoma model with CAR-T cells targeting CD19, over-expression of IDO depleted the anti-tumor activity of CAR-T cells and inhibited the cytokine-dependent expansion of CAR-T cells, cytokine secretion, and increased their apoptosis (55) (**Figure 1**). Production of lactic acid by tumor cells also inhibits the activity of CD8 T cells and NK cells. In detail, most tumors rely on glycolytic metabolism to sustain rapid cell growth through the enzyme lactate dehydrogenase-A that produces lactic acid. CD8 T cells and NK cells undergo a similar metabolic switch activating a glycolytic metabolism when they evolve from a naive to an activated state. However, highly glycolytic tumor cells are superior competitors for glucose and amino acids than CD8 T cells and NK cells. In addition, lactic acid production further suppresses nutrient uptake by CD8 T cells and NK cells, dampening their metabolic programs, leading to impaired activation of CD8 T cells and NK cells with the subsequent overcoming of immune surveillance by tumor cells (56).

### CAR-T Cells, a Strategy to Inhibit the Immunosuppressive TME and the Impact of Tumor Secretome

Adoptive cellular immunotherapy administering CAR-T cells has achieved outstanding and permanent responses in pediatric-B cell hematological malignancies with persistence of CAR-T cells over the years (26). However, in other hematological malignancies (15, 27) and solid tumors, results have been more inferior due to a short persistence of CAR-T cells and the barriers that CAR-T cells have to face in the TME, such as the impact of the tumor secretome. Fourth-generation CAR-T cells, termed armored or TRUCK CARs, are equipped with different features that can remodel the TME to enhance the activity of CAR-T cells.

Thus, a variety of armored CAR-T cells that secrete different cytokines have been developed. For instance, CART-19 cells that secrete IL12 show increased cytotoxicity and resistance to T-reg cell-mediated inhibition, better engraftment, and enhanced anti-tumor activity in models of B-cell malignancies (103) and ovarian cancer (104). Of note, severe adverse events were observed in a clinical trial with TILs secreting IL12 (105). Therefore, decreasing the amount of cytokines released by CART cells, in this case, IL12, could be modulated *via* different gene-expression cassettes, such as promoters in the CAR with inducible nuclear factor of activated T cells (NFAT) binding motifs (106). IL15 enhances the differentiation, homeostasis, and survival of T cells and NK cells. CART-19 cells secreting IL15 demonstrated increased expansion and efficacy, with decreased apoptosis and PD-1 expression, in models of Burkitt lymphoma (107). CAR-T cells secreting IL18 have caused increased M1-polarization in macrophages of the TME, depletion of M2-macrophages and T-reg cells (108), and recruitment of endogenous T cells (109). Nevertheless, as IL18 is pro-inflammatory, it has pathogenic roles in autoimmune diseases (110) and might also promote tumor progression, angiogenesis, immune escape, and metastasis (111). CAR-T cells secreting IL7 and CCL19 have also improved cell infiltration of dendritic cells (DCs) and survival of CAR-T cells (112). In addition, inhibition of TGFβ is achieved by co-expression in the CAR of a dominant-negative receptor for TGFβ that blocks TGFβ signaling, increasing proliferation and persistence of CAR-T cells in models of prostate cancer (113).

Armored CAR-T cells also avoid the negative impact of immune checkpoints. Thus, in lymphoma, the TME is marked by exacerbated lymphoid stroma activation and increased recruitment of follicular helper T cells, resulting from the disruption of the inhibitory checkpoint HVEM/BTLA. Secretion of HVEM by CAR-T cells binds BTLA avoiding this event (114). In addition, CAR-T cells that secrete anti-PD-L1 antibodies prevent T cell exhaustion and recruit NK cells to the tumors (115).

Furthermore, hypoxia is found in the TME and contributes to the rapid growth of tumor cells. Under hypoxia, glucose is fermented to lactate. The hypoxic TME also favors a glycolytic metabolism and increased lactate production, dampening T and NK cell effector functions and survival (59). Thus, armored CAR-T cells that secrete catalase (CAT-CAR) overcome hypoxia and reactive oxygen species (ROS) present in the TME (116). Another option to overcome these obstacles is to modify the CAR to express anti-oxidant factors such as N-acetylcysteine (NAC) that reduces DNA damage in CAR-T cells lowering activation induced-cell death in CAR-T cells (117).

### Decidual-Like NK Cells: An NK Cell Population Poorly Studied in Immunotherapy

The well-recognized anti-tumor activity of NK cells has led to many clinical studies administering either NK cells or CAR-modified NK cells, although results to date have shown mainly safety but not a high efficacy (18). These findings suggest the need to optimize NK cell anti-tumor efficacy. Here, we present studies that indicate that when NK cells arrive at the TME, events might happen that modify their killer activity.

In this regard, there are two main populations of NK cells in peripheral blood, the mature and cytotoxic NK with CD56^low^CD16^high^ expression, which constitutes 90% of NK cells, and the immature and immunoregulatory NK cells characterized by CD56^high^CD16^low/neg^CD25^+^ expression, which comprise approximately 10% of peripheral blood (PB)-NK (18, 19). A third transient population, known as decidual NK (dNK) cells, present at the fetal-maternal interface during the first months of pregnancy, representing 70% of immune cells in the decidua. dNK cells are also known as uterine NK (uNK) cells, as classically, uNK cells were detected by Dolichos biflorus agglutinin (DBA) lectin staining, where DBA+ cells were defined as dNK cells. Decidualization is triggered during blastocyst implantation and the menstrual cycle, characterized by a marked increase in dNK cells. dNK or uNK cells are a dynamic population, and their origin is not clear. A recent model proposed that there is a first wave of proliferation of tissue-resident NK cells in the pregnant uterus at the onset of the decidualization process. Then, a second wave involves the recruitment of conventional PB-NK cells during the placentation process (118, 119).

dNK cells are immune-tolerant and characterized by CD56^bright^CD16^−^CD9^+^CD49a^+^ and Eomes^+^ expression (120, 121). They are angiogenic, regulate trophoblast invasion and vascular growth during the placental developmental process and cooperate with other cells to serve as constructive elements during early pregnancy. dNK cells produce large amounts of proangiogenic factors, including VEGF, PlGF, CXCL8, IL-10, and angiogenin, critical for decidual vascularization and spiral artery formation (122). dNK cells also express chemokine receptors, including CXCR3, CXCR4, CCR1, CCR9, and the integrin ITGA3 (120), and through the interaction of HLA-G on fetal trophoblast cells with ILT2 and KIR2DL4, they secrete other growth-promoting factors, including pleiotrophin and osteoglycin (121). Moreover, interaction of soluble HLA-G with KIR2DL4 induces a pro-inflammatory response in dNK cells, activating their senescence with SASP secretion that promotes vascular remodeling and angiogenesis in early pregnancy (58).

This “nurturing” role of dNK cells during early pregnancy presents many homologies to NK cells infiltrated in different types of tumors. Thus, a subset of NK cells in non-small cell lung cancer, squamous cell carcinoma, or colorectal cancer turns into dNK-like cells inducing human umbilical vein endothelial cell migration and formation of capillary-like structures (36, 123–125). Various studies have tried to determine different factors during early pregnancy that might be responsible for this polarization of PB-NK cells into dNK-like cells. Results suggest that this polarization seems more specific for CD56bright than for CD56dim NK cells. Of interest, NK cells administered in immunotherapy treatments undergo an *in vitro* expansion that turns them into CD56^bright^ NK cells (17). Many of the factors responsible for this NK polarization are present in both the decidua and the TME, suggesting that these events occurring in the TME might impact the growth of tumor cells. In the next section, we detail the effect of secreted factors in the TME over the phenotype and polarization of NK cells.

### Impact of the Tumor Secretome in the PB-NK Cell Activity and Their Transition of Killer NK to dNK-Like Cells

Glycodelin-A is secreted in large amounts in the decidua and by tumor cells in malignancies, such as Non-Small Cell Lung Cancer (126), mesothelioma (127), ovarian cancer (128), and endometrial cancer (129). Glycodelin-A converts immunoregulatory CD56^bright^ PB-NK cells into dNK-like cells, an effect that does not occur for mature CD56^low^ PB-NK cells. This mechanism occurs through binding of Glycodelin-A to sialylated glycans on CD56^bright^ NK cells and causes enhanced expression of CD9, CD49a, and production of VEGF and IGFBP-1 that regulate endothelial cell angiogenesis and trophoblast invasion (57).

Soluble HLA-G is associated with bad prognosis in different tumors (130–134). Of interest, soluble HLA-G mediates polarization of PB-NK cells to dNK-like cells, with a senescent phenotype, secretion of growth factors, and reduced killer activity (58), thus, emerging as an essential target that can polarize the activity of NK cells.

**Figure 3:**
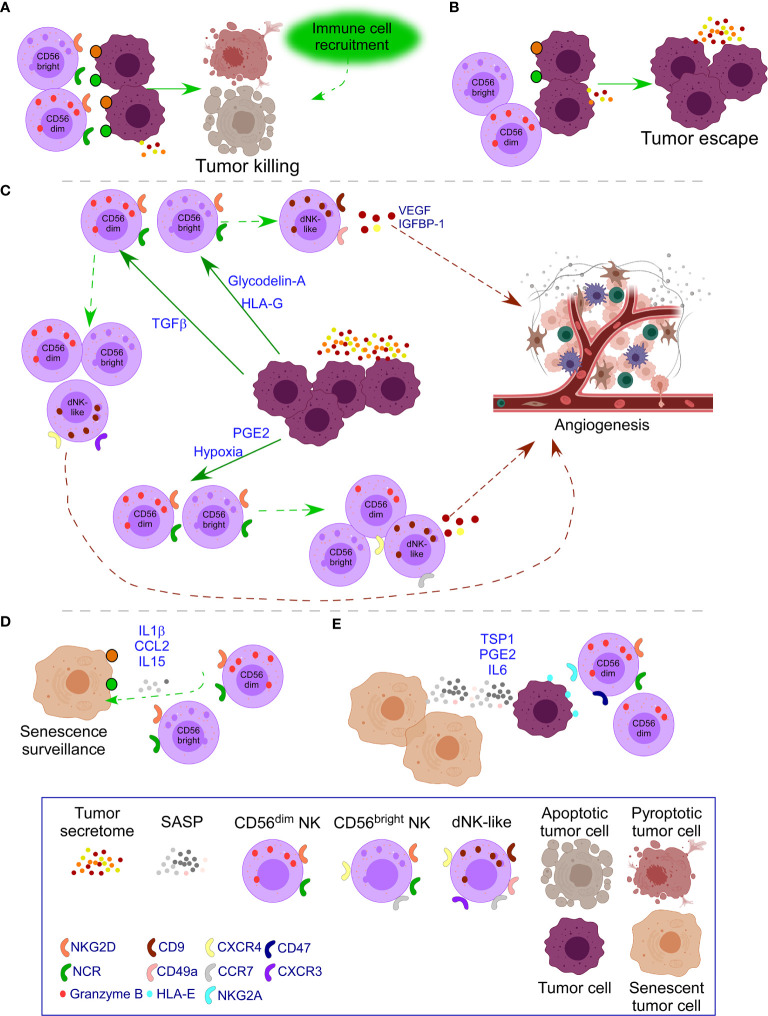
Impact of tumor secretome in NK cell activity. **(A)** In healthy conditions, NK cells recognize transformed cells through ligands of NKG2D and the family of NCR receptors (NKp30, NKp44, NKp46) which are over-expressed in transformed cells. Pro-inflammatory forms of cell death attract additional immune cells to cooperate in the killing. **(B)** In some cases, tumor cells down-regulate ligands for NK cell receptors or the tumor microenvironment (TME) causes down-regulation of activating NK cell receptors leading to tumor escape with additional secretion of tumor secretome. **(C)** When tumor escape occurs, increased tumor secretome leads to additional changes in NK cells. Specifically, release of Glycodelin-A and HLA-G converts immunoregulatory CD56^bright^ PB-NK cells into dNK-like cells. TGFβ converts both cytotoxic CD56^dim^ and CD56^bright^ NK cells into dNK-like cells; and down-regulates NK cell activating receptors limiting NK killer activity. PGE2 and hypoxia inhibit the expression of NK cell activating receptors and their functional maturation leading to suppressed NK cell cytotoxicity. Moreover, hypoxia, preserves immature CD56^bright^ NK cells with expression of receptors of dNK cells, resembling to dNK-like cells. In all cases, dNK-like cells will activate angiogenesis processes. **(D)** Emergence of senescent tumor cells leads to SASP secretion that attracts NK cells to mediate their clearance. **(E)** When the number of senescent cells increases, the SASP also does, leading to inhibition of NK cell activity, through mechanisms, such as the interaction of HLA-E with the inhibitory receptor NKG2A in NK cells and binding of TSP1 with CD47 that inhibit NK cell activity. PGE2 and IL6 in the SASP also down-regulate NK cell activating receptors. Moreover, therapy-induced senescence in established tumors down-regulates NK cell activating receptors on mature NK cells and their ligands on tumor cells.

TGFβ secretion can be beneficial at early stages and detrimental at late-stage tumor development by remodeling the TME to favor tumor growth (130, 135). TGFβ converts both cytotoxic CD56^dim^ and CD56^bright^ PB-NK cells into dNK-like cells (36, 37) (**Figure 3**). Moreover, IL15 and IL18 added to TGFβ enhance the impact on the polarization of PB-NK cells toward a dNK cell phenotype with increased expression of CD9, CD49a, ITGA3, and CXCR4 (38). Of interest, as previously mentioned, IL15 and IL18 are beneficial for CAR-T cells (107–109), suggesting the negative role of these cytokines when TGFβ is added. Additional effects of TGFβ over NK cells include down-regulation of NKP30, NKG2D (39), and DAP10 and, consequently, NKG2D (40) inhibiting NK cell function (**Figure 3**). Of interest, this dual role of TGFβ in the TME is observed when at low doses facilitates NK cell recruitment to the tumor by up-regulating CXCR4 and CXCR3, markers of dNK; and at high doses, down-regulates NKp30, limiting NK killer activity (41).

PGE2 secretion in thyroid cancer and melanoma inhibits the expression of NKG2D, NKp44, NKp30, and TRAIL on PB-NK cells and their functional maturation leading to suppressed NK cell cytotoxicity (10, 52) (**Figure 3**). PGE2 release by cancer-associated fibroblasts in melanoma down-regulates NKp44 and NKp30 leading to NK cell inhibition (53). Soluble PVR and Nectin-2 released by tumor cells bind to TIGIT on NK cells inhibiting NK cell cytotoxicity (50).

Hypoxia is another factor present in both the decidua and the TME. Hypoxia in the TME avoids the ability of NK cells to upregulate NKp46, NKp30, NKp44, and NKG2D in response to activating cytokines (60) and degrades NK cell granzyme B by autophagy (61), impairing the ability to kill and promoting immune evasion (**Figure 3**). Moreover, exposure to a combination of hypoxia, TGFβ, and 5-aza-2′-deoxycytidine, results in the polarization of PB-NK cells to dNK-like cells. These changes are more pronounced when all the factors are together and lead to the expression of CD9, CD49a, chemokine receptors, and VEGF secretion that leads to dNK-like cells with capacity to promote invasion of trophoblast cell lines and reduced cytotoxicity. Significantly, these parameters are reversed after returning to normal conditions, indicating the plasticity of immune cells (37). Exposure of PB-NK cells to hypoxia also causes reduced NK cell ability to release IFNγ, TNFα, GM-CSF, CCL3, and CCL5, and preservation of immature CD56^bright^ NK cells expressing CCR7 and CXCR4, resembling dNK-like cells (62).

The impact of these tumor secreted factors occur mainly on CD56^bright^ PB-NK cells, and NK cells used in immunotherapy undergo an *in vitro* expansion that turn them into CD56^bright^ NK cells (17). These events suggest that in cases that NK cells do not achieve complete removal of tumor cells they might have polarized into dNK-like cells. Therefore, monitoring these changes in immunotherapy NK cell studies will provide relevant information to improve the clinical outcome of patients.

### Role of Macrophages in Immune Surveillance

Macrophages are innate immune cells with high plasticity which traditionally, have been classified as two extremes being either pro-inflammatory (M1: activated) or anti-inflammatory (M2: alternatively activated). M1 inhibits cell proliferation and causes tissue damage, while M2 promotes cell proliferation and tissue repair. M1 and M2 enable Th1, and Th2 responses, respectively, and consequently, Th1 and Th2 cytokines regulate their activity. Thus, M1 responds to IFN-γ, TNF-α, and TLR4 activation, and M2 to IL-4 and IL-13 (136). However, macrophages present high plasticity and convert to a wide variety of subpopulations depending on the stimuli they receive from the TME (63, 137). Macrophages represent the largest population of all infiltrating leukocytes in the tumor (138), where tumor-associated macrophages (TAMs), which present an M2-like phenotype, are considered highly responsible for tumor progression, and many studies have focused on trying to polarize M2-like macrophages to M1 (139). However, M2 are the macrophages with the highest phagocytic activity against apoptotic tumor cells (140), suggesting that removing this activity might also be detrimental. Therefore, efforts should be directed to preserve M1 macrophage activity while also enhancing the phagocytic activity of M2 macrophages. Here, we will pay special attention to the phagocytic function of M2 macrophages to remove tumor cells and how secreted molecules in the TME can polarize macrophages to an M2-like or M1 phenotype.

**Figure 4:**
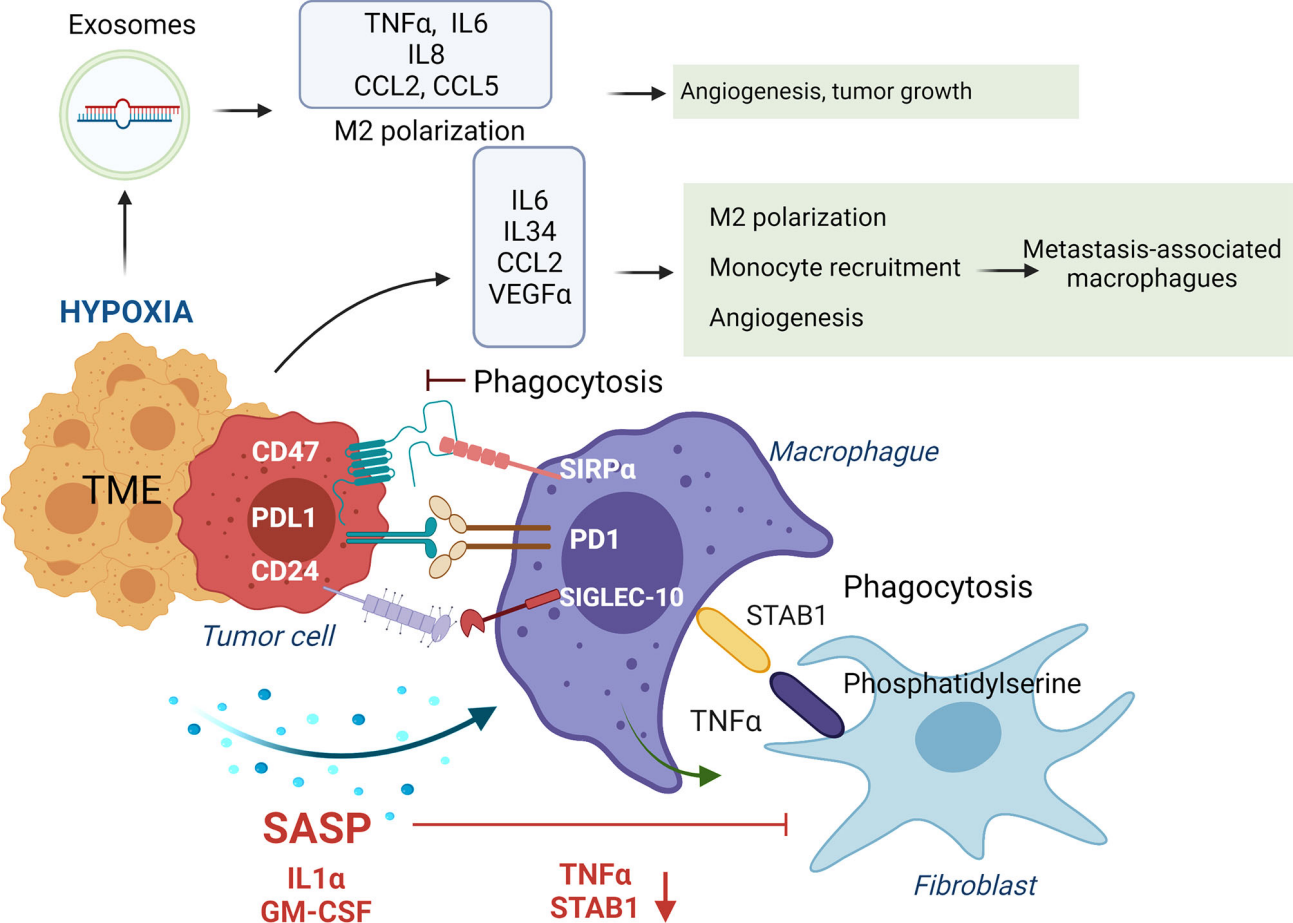
Impact of tumor secretome in the phagocytic activity of macrophages. In healthy conditions macrophages phagocyte transformed cells and senescent fibroblasts to maintain tissue homeostasis. Normally, macrophages, through release of TNFα, induce apoptosis in senescent fibroblasts, leading to expression of phosphatidylserine in their surface, which is recognized by STAB1 on macrophages to promote their phagocytosis. In advanced stages of senescence, phagocytic activity of macrophages is inhibited by over-expression of ligands of immune-checkpoints (CD47, PDL-1 and CD27) that interact with their receptors on macrophages (SIRPα, PD1 and SIGLEC-10). Moreover, SASP factors, including IL1α and GM-CSF, down-regulate STAB1 and TNFα expression, avoiding the phagocytosis of senescent fibroblasts by macrophages. In addition, IL6, IL34, CCL2 and VEGFa secretion in the TME, induce M2 macrophage polarization and recruitment of inflammatory monocytes that polarize to metastasis-associated macrophages that in summary promote tumor growth. Hypoxia in established tumors also promotes the release of exosomes containing the miRNAs miR-301a-3p and miR-21 that promote M2 polarization, and TNFα, IL6, IL8, CCL2 and CCL5 secretion impacting in higher angiogenesis, and tumor growth.

Phagocytosis of tumor cells by macrophages is performed after recognizing “eat me” or don’t eat me” signals that will or will not trigger phagocytosis. “Eat me” and “don’t eat me” signals act as ligands for phagocytic receptors that will or will not trigger the engulfment of the target. Different studies have shown the beneficial impact in tumor regression of inhibiting these “don’t eat me” signals. For instance, CD47 expression in small-cell lung cancer cells engages SIRPα on macrophages inhibiting their phagocytic activity, which is recovered with an anti-CD47 (75). Moreover, inhibition of CD47 in tumor cells promoted their phagocytosis and the anti-tumor activity of CD8 T cells while inhibiting T-reg cells (141). Blocking PD-1 expressed in TAMs or M2-like macrophages increases macrophage phagocytosis and reduces tumor growth (76) (**Figure 4**).

Furthermore, anti-PD-L1 treatment reverses the immunosuppressive status of the TME and enhances specific T cell anti-tumor effects in murine models of cancer (142). Interaction of β2M subunit of HLA-I in tumor cells with LILRB1 on macrophages protects tumor cells from phagocytosis by TAMs, and disruption of this interaction potentiates phagocytosis of tumor cells (77). In ovarian cancer and triple-negative breast cancer, tumor cells evade clearance by macrophages through over-expression of CD24 that interacts with Siglec-10 in TAMs, and its blockade augments the phagocytosis of CD24-expressing tumors leading to a reduction of tumor growth (78). Dectin-2, a C-type lectin receptor in macrophages resident in the liver (Kupffer cells), promotes phagocytosis of cancer cells, avoiding liver metastasis (143) (**Figure 4**).

Phagocytosis requires an intimate contact of the macrophage and the target, where the glycocalyx, a layer that surrounds the plasma membrane containing glycolipids, glycoproteins, and surface-associated glycosaminoglycans, acts as a barrier for these contacts. The size and charge of this glycocalyx can be modified and modulated by enzymes or other molecules present on the TME to promote phagocytosis (144). Of interest, we described that NK cells release histones that bind to and degrade the syndecans on the glycocalyx of multiple myeloma cells (145), suggesting that by doing this, NK cells might also promote the phagocytosis of tumor cells by macrophages, an event observed during fungal infection (146).

Molecules secreted in the TME will also impact promoting an anti-inflammatory or pro-inflammatory environment that will polarize macrophages into TAMs/M2-like or M1 phenotypes. For instance, IL6 secretion in the TME induces M2 macrophage polarization in colorectal cancer models (66). The release of oncostatin M in the TME is involved in M2 polarization *via* mTOR signaling complex 2-Akt1 (67). At breast cancer, the release of CCL2 by tumor cells recruits inflammatory monocytes that polarize to metastasis-associated macrophages, which secrete CCL3, promoting lung metastasis (68). IL34 secretion by tumor cells binds to CSF1R in macrophages and polarizes them to M1 and M2 (147). Also, IL34 contributes to osteosarcoma growth by increasing the neo-angiogenesis and recruitment of M2 macrophages (69). In a skin carcinogenesis model, VEGF-A expression on tumor cells with IL10 and IL4 secreted by tumor cells and macrophages, respectively, induced M2 polarization that promoted tumor growth (70). Release of the proteglycan versican by lung carcinoma cells activates macrophages to release TNFα enhancing growth of tumor cells (71) (**Figure 4**).

Tumor hypoxia, a feature of the TME, promotes ID4 expression in cancer cells which, through VEGF, activates increased expression of granulin in macrophages, conferring increased angiogenic potential (63). In pancreatic cancer cells, the presence of hypoxia promotes the release of exosomes containing the miRNA miR-301a-3p that binds to TLR macrophages receptors, promoting M2 polarization, TNFα, and IL6 production, creating a pro-metastatic environment (64). Hypoxia induces CXCL12 and CXCR4 expression, which modulate the migration of monocytes, monocyte-derived macrophages, and TAMs (65) (**Figure 4**). Of interest, when hypoxia is absent in tumor cells, TAMs can enhance tumor hypoxia and glycolysis (148), being both features that promote tumor aggressiveness (149).

Exosomes released in liver tumors bind to macrophages through exosome integrins and prepare the pre-metastatic niche (150). In pancreatic ductal adenocarcinomas, tumor-derived exosomes with macrophage migration inhibitory factor are taken by Kupffer cells causing TGFβ secretion. Consequently, a fibrotic microenvironment emerges that recruits macrophages, creating a liver pre-metastatic niche (72). Of interest, the release of ST2 in Rab37 exosomes skewed M1 macrophage polarization leading to reduced tumor growth in models of lung cancer. Moreover, lung cancer patients with low Rab37, low soluble ST2, and low M1/M2 ratio presented worse overall survival (73). SNAIL, a transcription factor expressed during epithelial-mesenchymal transition, activates the production of tumor-derived exosomes containing miR-21 that will be phagocyted by monocytes leading to M2 macrophages, secretion of IL6, IL8, CCL2, and CCL5 impacting in higher angiogenesis, and tumor growth (74) (**Figure 4**).

## Acquisition of Therapy Induced-Senescence (TIS) After Chemotherapy and Its Impact on Immune Cells

Studies have demonstrated that chemotherapy treatment can lead to acquired resistance and the emergence of more aggressive tumor cells. In this regard, the tumor secretome is shaped by chemotherapy treatment that will impact the immune response and increase tumor aggressiveness. For instance, in breast cancer, IL6 release after treatment converts differentiated tumor cells to cancer stem cells through the IL6-JAK1-STAT3 pathway (151). In non-small cell lung cancer, cisplatin induces IL6 secretion that increases tumor progression and resistance to treatment through up-regulation of anti-apoptotic proteins and DNA repair associated genes (152). Paclitaxel enhances IRE1 RNase activity that leads to the production of IL6, IL8, CXCL1, GM-CSF, and TGFβ2 in breast cancer cells contributing to the expansion of tumor-initiating cells (153). Doxycycline treatment in squamous cell carcinoma leads to TGFβ secretion that activates the TGF-β/SMAD pathway increasing tumorigenic potential (154). Treatment with kinase inhibitors causes secretion of positive mediators of the AKT pathway, including IGF1, EGF, ANGPTL7, and PDGFD, accelerating the expansion and dissemination of drug-resistant clones (155). Docetaxel induces secretion of extracellular vesicle-encapsulated miRNAs, including miR-9-5p, miR-195-5p, and miR-203a-3p, which down-regulate the transcription factor ONECUT2, leading to up-regulation of stemness-associated genes, that stimulate cancer stem-like cells and resistance to therapy in breast cancer (156).

In addition, chemotherapy and radiotherapy treatments trigger a premature state of senescence in tumor cells termed “therapy-induced senescence” (TIS) that will shape the tumor secretome (29, 157). TIS can reactivate the cell cycle and bring on cancer daughter cells that survive therapy more transformed than the original population (158, 159). This secretome is unique because it is induced by senescence, being termed senescence-associated secretory phenotype (SASP). SASP includes various cytokines, chemokines, growth factors, and matrix metalloproteinases, such as IL1α, IL1β, IL6, IL8, CXCL1, CCL2, VEGF, and CXCR2 (29, 160, 161), that interfere with the paracrine activity of senescent cells. Of interest, SASP released by tumor cells after TIS induces transmission of senescence to non-senescent neighboring cells (162, 163). The SASP can foster an immunosuppressive environment favoring metastasis (160), and on the other side, attracts immune cells including macrophages, neutrophils, and NK cells to remove senescent cells, a process known as “senescence surveillance” (164–167).

Moreover, cancer is associated with aging. A physiological consequence of aging is the development of immunosenescence due to a functional degradation of the thymus, resulting in decreased functional naïve CD4 and CD8 T cells and a peripheral oligo-clonal expansion of memory T cells. These events provide a contracted T cell antigen receptor (TCR)-repertoire diversity with secretion of SASP (29, 168). Immunosenescence associated with aging also occurs due to exposure to virus infections or chronic inflammation (169); and additional factors such as nutrition, sex, genetics, previous diseases, or tumors (170, 171). Therefore, the immune cells of elderly cancer patients will probably be already senescent; and moreover, SASP secretion by senescent tumor cells after chemotherapy will accelerate this immunosenescence process (171). Here, we will mention some SASP factors released by tumor cells that impact the anti-tumor immune response.

### Impact of the SASP in T Cells and Immunosenescent T Cells

Studies have shown a significant accumulation of senescent T cells in certain types of cancer patients (172), and that tumor SASP induces T cell senescence leading to suppression of responses of naïve/effector T cells (173), suggesting that this might be a strategy used by malignant cells to evade immune surveillance. Transformed senescent T cells are in cell cycle arrest and develop significant phenotypic alterations, such as down-regulation or loss of CD27 and CD28.Through SASP factors including pro-inflammatory cytokines or inhibitory molecules like IL10 or TGFβ, senescent T cells will amplify the immunosenescence process. Moreover, the development of exhaustion with high expression of immune checkpoints, such as TIM-3 and other co-inhibitory receptors as CD57 or KLRG-1, will promote replicative senescence of T cells (174).

TGFβ1 and TGFβ3 are early SASP factors that regulate thymic T cell homeostasis, inhibit cytotoxic T cell proliferation, and promote T-reg generation (175). Tumor senescent cells up-regulate NOTCH1 and drive a TGFβ-rich secretome that suppresses the release of a pro-inflammatory SASP and contributes to the transmission of senescence through cell-cell interaction *via* NOTCH-JAG1 pathway. Of interest, NOTCH1 inhibition recovers the secretion of pro-inflammatory cytokines, promoting lymphocyte recruitment and senescence surveillance (176). Senescent cells, after genotoxic stress, secrete IL6 and IL8 that promote epithelial-mesenchymal transition, increasing tumor cells’ invasiveness. Moreover, IL6 recruits myeloid cells that inhibit T cell responses (177).

MAPK signaling is a relevant pathway that controls T cell senescence (178) through activation of p53, p21, and p16 (179). Recent research demonstrated that tumor-derived T-reg cells exhibit an accelerated glucose uptake, competing with effector T cells for glucose through TLR8 signaling, leading to MAPK activation, which induces T cell senescence (180). Another study showed that T-reg cells, through p38, ERK1/2 signaling, p16, p21, and p53 induce senescence in responder naïve and effector T cells. This event is reverted by the block of TLR8 signaling and or by specific ERK1/2 and p38 inhibition (181). Moreover, the p53 isoforms Δ133p53 and p53β regulate proliferation and senescence in human T lymphocytes. Thus, decreased Δ133p53 and increased p53β expression in healthy individuals and lung cancer patients associated with age-dependent accumulation of senescent CD8 T cells (182).

The hypoxic TME leads to the accumulation of adenosine and tumor-derived cAMP. This cAMP is a SASP factor that induces T cell senescence in naïve/effector T cells. Of interest, activation of TLR8 signaling in tumor cells reverses this event resulting in enhanced anti-tumor immunity (183). Moreover, the accumulation of adenosine in the TME also inhibits the anti-tumor activity of T cells through the adenosine receptor A2AR, which in healthy conditions regulates immune cells protecting from inflammatory damage (184).

### CAR-T Cells

Whereas the immunosenescence process has been widely studied in T cells, there is a lack of information related to CAR-T cell senescence. It could be exciting to delve into the mechanisms of senescence of this type of cells to find pathways to inhibit senescence without impacting their anti-tumor activity. Specifically, CAR-T cells undergo a significant *in vitro* expansion (185) to obtain enough CAR-T cells to treat the patients. This expansion might impact the development of senescence due to continuous *in vitro* proliferation. Moreover, the transfer of senescence from tumor cells in the TME mediated by cell-cell contact or through factors present in the SASP will impact CAR-T cell activity. CAR-T cells can be engineered to avoid these events. Thus, recently, CAR-T cells have been used as senolytic agents in lung adenocarcinoma to remove chemically induced senescent cells by targeting the urokinase-type plasminogen activator receptor (186).

A tempting option that could be tested is to reverse early-stage senescent CAR-T cells by blocking critical mediators of this process, such as proteins involved in the DDR, p38, p53, p21, or ATM (187). However, these changes could also decrease T cell functionality by impacting other relevant functions. For instance, p38 is involved in the induction of senescence and IFNγ and TNFα secretion (188), and its inhibition have diminished these cytokines in different inflammation or virus infection models (189, 190). Moreover, blockage of DDR and p53 involves a risk of DNA damage on T cells that might induce malignancy (191).

### SASP Impact in NK Cells and Senescence Surveillance

NK cells have an essential role in the senescence surveillance of tumor cells. Senescence surveillance is initiated by the SASP that activates immune cells to clear senescent cells preventing tumor initiation (167), where both macrophages and NK cells have an important task (32, 192, 193). Proteins present in the SASP, such as CCL2, attract PB-NK cells to remove senescent cells through NKG2D (194). Of interest, this role of PB-NK cells removing senescent cells is also observed by decidual uterine NK cells to control embryo implantation. Specifically, dNK cells after being activated by IL15, present in the SASP, target and clear decidual cells that became senescent in an IL8 dependent manner. This mechanism of NK cells is mediated through granule exocytosis and involvement of NKG2D (195).

SASP secretion by senescent tumor cells up-regulates HLA-E, the ligand of the inhibitory NKG2A NK receptor (196), and cleave NKG2D ligands inhibiting NK cell activity (197).

Soluble Thrombospondin-1 (TSP1), released in the SASP, is involved in Ras-induced senescence (198). Moreover, TSP1 released by tumor cells binds CD47 on NK cells inhibiting its activity (199). CD47 is described as a relevant modulator of NK cell function in virus infection (200). Of interest, after TIS, binding of soluble TSP1 to CD47 causes emergence of tumor-resistant cells and metastasis in triple-negative breast cancer (201), and inhibits anti-melanoma NK cell activity with reduced granzyme B and IFNγ production (202) (**Figure 3**).

IL1β is another crucial molecule present in the SASP with a relevant pro-tumor activity (203). In detail, IL1 signaling controls the SASP production (204), and transmission of IL1β to neighboring cells induces cell senescence (205, 206). A dual role for IL1β is observed in NK cell activity. For example, IL1β is required by CD56^bright^ NK cells to produce IFNγ (207) to activate pyroptosis, necessary for the anti-microbial (208) and anti-tumor (145) activity of NK cells. In addition, IL1β released by M1 macrophages increases NK cell cytotoxicity up-regulating NKp44 and NKG2D and triggering IFNγ production by NK cells. Of interest, these IL1β-primed NK cells can reverse M2 macrophage polarization (209). On the other side, a negative impact of IL1β has been described over NK activity. Thus, tumor-derived IL1β induces accumulation of MDSCs that impair NK cell development and functions (210). Moreover, a higher secretion of IL1β in endometrial cancer patients compared to healthy tissues correlates with infiltrating CD56^bright^ NK cells in the tumor with exhausted phenotype, indicated by TIGIT and TIM3 expression (211).

IL6 and IL8, present in the SASP, favor the acquisition of migration/invasion and stem-like features, increasing tumor aggressiveness in breast cancer cells (212, 213). Moreover, IL6 also inhibits NK cytotoxic activity by down-regulating perforin and Granzyme B (214). In esophageal squamous cell carcinoma, tumor cells activate the STAT3 pathway on NK cells through IL6 and IL8, leading to down-regulation of NKp30 and NKG2D on NK cells and tumor progression (215) (**Figure 3**). In addition, increased levels of IL6 in the peritoneal fluid of endometriosis patients reduced the cytolytic activity of NK cells with down-regulation of granzyme B and perforin (216). IL8 activates and recruits immune cells (217) but also has tumor-promoting functions (218). IL8 is produced by CD56 bright NK cells (219), and stimulation with IL18 and IL12 induces higher IL8 production by NK cells (220).

PGE2 secretion, present in the tumor secretome, inhibits NK cell activity (10, 52, 53). Moreover, PGE2 is also present in the SASP at early tumorigenesis stages, secreted by COX-2, a critical regulator of the SASP, and promotes senescence surveillance (221) (**Figure 3**).

Senescent cells show high ROS levels and lactate production that induce and maintain cell senescence (222, 223). ROS can present contradictory effects on the activity of NK cells. Specifically, lactate production by metastatic colorectal cancer cells induces mitochondrial stress, increased ROS, and apoptosis in NK cells (224). On the other side, ROS is required for the anti-tumor activity of NK cells (225). Moreover, TIS up-regulates NKG2D ligands (MICA, MICB, and PVR) in an oxidant-dependent manner, resulting in enhanced NK cell activity against myeloma cells (226). This up-regulation of NKG2D ligands upon oxidative stress was also observed in colon carcinoma cells, leading to improved NK cell killing (227). However, in established tumors, ROS down-regulates NKp46 and NKG2D on mature CD56^dim^ NK cells inducing suppression of NK activity against melanoma (228) and acute myeloid leukemia cells (229). Of interest, we previously observed that cord blood-derived NK cells reduce ROS levels in multiple myeloma cells (230). This negative role of ROS in tumors has led to antioxidant treatments in cellular immunotherapy studies. For instance, as previously mentioned, in solid tumors, CAR-T cells modified to express the enzyme catalase presented an anti-oxidant capacity to protect bystander T cells and NK cells (116).

All these studies suggest the beneficial and detrimental role of the SASP at early and late stages of tumorigenesis, respectively. As high levels of SASP inhibit NK cell activity, a strategy to treat advanced cancer patients with cellular immunotherapy, could be to administer senescence inhibitors to decrease the number of senescent cells. Once reduced levels of SASP are achieved, immune cells could be administered, that would be attracted to remove the remaining senescent tumor cells.

### Macrophages

Macrophages are attracted and stimulated by SASP factors including MCP-1, MIP-1α, and GM-CSF to remove senescent cells (231). Macrophages are also affected by age-related immunosenescence and the consequences of inflammaging, a chronic inflammation occurring with aging, leading to macrophage dysfunction. Increased levels of A20, a suppressor of the NFκB and MAPK signaling, mediated this dysfunction, leading to poor NFκB and MAPK activation following TLR stimulation (232).

There is a disparity in the impact of TIS and the SASP in macrophage polarization and their phagocytic activity. Thus, in a model of skin aging, macrophage activity is inhibited when there are a high number of senescent cells (233). Specifically, through TNFα release, macrophages induce apoptosis in senescent fibroblasts, leading to the expression of phosphatidylserine on their surface. Phosphatidylserine is recognized by the STAB1 receptor on macrophages to promote their phagocytosis. However, SASP factors, including IL1α and GM-CSF, down-regulate STAB1 and TNFα expression, avoiding the killing and phagocytosis of macrophages, with no impact observed in the macrophage polarization (233).

In a model of thyroid cancer, monocytes exposed to conditioned media from senescent thyrocytes and thyroid tumor cells, undergo M2-like polarization displaying tumor-promoting. These events were related to the production of PGE2 (234). In liver fibrosis and cirrhosis, hepatic stellate cells made senescent by carbon tetrachloride treatment produce cytokines that recruit M1 macrophages, promoting a tumor-suppressive environment. However, in the absence of p53, a promoter of senescence, the released secretome induces M2 polarization, enhancing premalignant cells’ proliferation (235). In a model of pancreatic cancer with oncogene-induced senescence, the SASP factor CXCL1 activates CXCR2 that leads to recruitment of M1 macrophages, inhibiting carcinogenesis. However, oncogene-induced senescence and SASP are bypassed at late stages, and M2 macrophages are recruited to enhance the proliferation of the transformed pancreatic cancer cells (236).

## Impact of the Type of Cell Death Activated in the Tumor Secretome

Finally, we call the reader’s attention to the type of cell death activated in tumor cells after the attack of immune cells in adoptive cellular immunotherapy. Inflammatory forms of cell death include pyroptosis, which activates the NLRP3 inflammasome, leading to IL1β production (237). As previously mentioned, IL1 signaling controls the SASP production (204). Of interest, CAR-T cells and NK cells used in adoptive cellular immunotherapy activate pyroptosis when they encounter the tumor cell (145, 238). These events suggest that the consequences of this IL1β release should be considered. Expressly, inflammasome activation and pyroptosis execution represent a double edge-sword in cancer immunotherapy, as on one side, pyroptosis executes cell death. On the other side, pyroptosis and IL1β production activate multiple signaling pathways and inflammatory mediators that promote tumor growth and metastasis in cancer models (239, 240), triggering TAMs to boost tumor angiogenesis (241). Moreover, the role of pyroptosis is highly relevant to attracting other immune cells through IL1β and IL18 secretion. These events are observed in microbial infections, where pyroptosis attract immune cells to kill the previously trapped pathogen and remove the infected cell (208, 242). In adoptive cellular immunotherapy, removing dead tumor cells after being killed by immune cells is required, suggesting an advantage of pyroptosis in this context.

## Conclusions

To conclude, adoptive cellular immunotherapy has emerged as a promising treatment to treat cancer patients in the last years. However, results still need to be improved in a variety of malignancies. Immune cells present a high capacity of plasticity when they receive stimuli from secreted molecules in the TME. Thus, if immune cells do not remove tumor cells, tumor secretome could modify their killer activity to an angiogenic or immunosuppressive one. A highly relevant aspect that needs to be considered to avoid these events is an efficient removal by macrophages of dying/dead tumor cells after the attack of immune cells, such as NK cells or CAR-T cells. Of interest, NK cells present additional functions to their classic killer activity that might help in this tumor cell surveillance. Inflammatory forms of cell death activated by *in vitro* expanded immune cells might also impact these processes. In summary, to achieve complete and permanent responses in cancer patients treated with adoptive cellular immunotherapy, all these aspects together need to be considered and count on the activity of the whole immune response and not just one immune cell population.

## Author Contributions

All authors listed have made a substantial, direct, and intellectual contribution to the work, and approved it for publication.

## Funding

This research was funded by Fondos Feder with a grant of the Institute of Health Carlos III, grant number PI20/00991.

## Conflict of Interest

The authors declare that the research was conducted in the absence of any commercial or financial relationships that could be construed as a potential conflict of interest.

## Publisher’s Note

All claims expressed in this article are solely those of the authors and do not necessarily represent those of their affiliated organizations, or those of the publisher, the editors and the reviewers. Any product that may be evaluated in this article, or claim that may be made by its manufacturer, is not guaranteed or endorsed by the publisher.
